# The relationship between the relative age effect and performance among athletes in World Handball Championships

**DOI:** 10.1371/journal.pone.0230133

**Published:** 2020-03-26

**Authors:** Alfonso de la Rubia, Christian Thue Bjørndal, Joaquín Sánchez-Molina, José María Yagüe, Jorge Lorenzo Calvo, Sergio Maroto-Izquierdo

**Affiliations:** 1 Department of Sports, Faculty of Physical Activity and Sports Sciences, Universidad Politécnica de Madrid, Madrid, Spain; 2 Department of Coaching and Psychology, Norwegian School of Sport Sciences, Oslo, Norway; 3 Faculty of Physical Activity and Sports Sciences, Universidad Europea, Villaviciosa de Odón, Madrid, Spain; 4 Faculty of Physical Activity and Sports Sciences, Universidad de León, León, Spain; University of Illinois at Urbana-Champaign, UNITED STATES

## Abstract

This study examines the relative age effect (RAE) and its impact on the performance of elite male (n = 3,358) and female (n = 3,273) handball players in the U-19 (n = 2,188), U-21 (n = 2,031), and senior (n = 2,412) categories of the 2013/2014, 2015/2016 and 2017/2018 World Handball Championships organised by the International Handball Federation (IHF). The distribution of the players´ birth dates (by quartile: Q, and semester: S) by categories, handball playing positions, and membership of continental federations, were analysed for each of the three two-year periods in which the World Handball Championships took place. Differences between the observed and expected birth dates were tested using a chi-square goodness of fit test, and subsequent calculations were tested using odds ratios. The Spearman’s correlation coefficient was applied to test the correlation between the relative age of the athletes and (their) performance parameters. The results revealed a prevalence of the RAE on both male and female in the U-19 and U-21 categories (*p<0*.*001*), and a stabilisation of the RAE throughout the period analysed (2013/14-2017/2018). The RAE was not found in the female senior category (2013, 2015 and 2017) or male senior category (2013) (*p>0*.*05*). All playing positions were impacted by the RAE (*p<0*.*001*), and especially strong effect sizes were noted for male pivots (*Vc = 0*.*66/0*.*68/0*.*60*) and female center backs (*Vc = 0*.*71/0*.*65/0*.*71*). In our analysis of the handball continental federations, the RAE was found in all regions, except Oceania (*p>0*.*05*). The RAE also affected athlete performance: the oldest athletes played more minutes and achieved better performances. The RAE was associated with the final placement of the teams in each championship category, except in the male youth category. The findings clearly demonstrated that the RAE has a significant impact on the selection of male and female athletes to international competitions and the performance of both men and women in international handball. The potential implications of these findings for policy and practice are discussed.

## Introduction

Successful talent identification and development (TID) systems are regarded by policy makers as vital to achieving success in adult elite sports, and are based usually on the selection of athletes for special activities, teams and/or competitions [1]. The introduction of international competitions at the youth elite level, for example, has become an important part of identifying, fostering and developing sporting talent in elite development systems [2], and serves as a mechanism for recognising sporting excellence [3]. However, attempts to identify sporting talent often conflate athletes´ future potential with their current performance [4]. Selection-based models frequently fail to recognise that the development of children and youth are strongly related to maturation and are highly variable [5], and the models tend to favour early developers [6]. While an athlete´s maturation does not necessarily correspond with her or his chronological age at the individual level, relatively older athletes tend to be more mature than relatively younger athletes at the aggregated level [7]. This is known as the relative age effect (RAE), and large scale-studies have shown that this effect impacts sporting careers by systematically increasing the number of opportunities made available to relatively older athletes [8].

In 2009, Cobley et al. [9] conducted a meta-analysis of the impacts of the RAE across sports, in a study which included 253 samples, and a total of 130,108 athletes. The analysis confirmed that the RAE had an impact in most sports, both on an individual level and on a team level: for every two subjects born in the last quartile of an age group, more than three of the first quartile of the same age group participated in the same sporting context. The RAE appears to have a large impact on the selection processes associated with TID systems in team sports such as ice hockey [10], basketball [11,12], football [13–15], rugby [16], Australian football [17], and handball [18,19]. However, recent research in women´s sports has reported more diverse findings, and the impact of the RAE in these seems to be less extensive [20]. The lower prevalence of the RAE reported in women’s sport may be due to a variety of factors, including the number of active participants, specific social and cultural contexts, the level of the competitions, and the popularity of the sports [21].

Studies focusing on the impacts of the RAE on the selection of athletes to TID systems in handball have also reported mixed findings. Schorer et al. [22], for example, examined selected vs. non-selected players in Germany´s national talent development system, and compared a sample of 475 U-17 handball players (237 males and 238 females). The results did not show an overrepresentation of players born in the first quartile compared to those born later, or between those who were selected, and those who were not. In contrast, Schorer et al. [23] demonstrated the historical impact of the RAE on men and women in Germany´s national system of talent development during the handball seasons from 1993 to 2007. However, the impacts of the RAE were not evident at the professional handball level in Germany. Similar mixed findings were reported by Sánchez-Rodríguez et al. [24] in their study of 161 male players from the Spanish national handball teams (promising, youth, junior and senior), and by Gómez-López et al. [25] in a study of 479 adolescent handball players who participated in the Spanish Regional Handball Championships during the 2015–16 season.

Wrang et al. [26] confirmed that relatively older players in Danish international handball teams were over-represented at the U-19, U-21, and senior levels, compared to relatively younger players. The study also noted that the impacts of the RAE appeared to decrease as the age of the players and the competition levels increased. The authors noted that relatively younger players of the same age cohort (U-19) were more likely to be selected in the Danish talent system, compared to relatively older players. Bjørndal et al. [27] reported notably skewed birth date distributions among the men and women selected to the U-19 and U-21 Norwegian international handball teams. However, at the senior level, the birth date distributions appeared to be uniform.

Only one study has compared the impact of the RAE in different countries. In a study of athletes selected to international youth competitions in handball, Fonseca et al. [28] included 383 youth male handball players from the 24 countries participating in the 7^th^ Men’s Youth World Championship held in 2017. Findings showed the prevalence of the RAE in the population sample but found no relationship between the age of the players and the success of each team–measured as a team´s championship ranking. Surprisingly, it was concluded that the selection bias shown at the youth international team level, did not influence the long-term sporting achievements of the athletes.

To the best of our knowledge, no studies have investigated the prevalence of the RAE at the international senior, U-21 and U-19 levels, or male/female team categories in handball. Other studies of the RAE in handball have been limited to specific geographical areas, specific countries or handball federations, or have focused on particular categories, male/female categories, years/seasons, or championship levels. Moreover, the extent to which the RAE affects future performance, after an athlete´s initial selection, remains unclear. The aim of this study, therefore, is to analyse the impacts of the RAE across different countries on the selection of athletes to international youth competitions, and the influence of the RAE on athlete and team performance in the U-19, U-21, and senior categories during the 2013/14, 2015/16 and 2017/18 World Handball Championships. The importance of this research lies in the need to interrogate the systematic attempts to facilitate talent development in sports, and helps to question whether the current procedures used in talent development systems are evidence-based. A lack of in-depth analysis of the RAE prevents a full understanding of its potential impacts and unintended consequences. It may also lead to the identification of other, more effective and appropriate practices [29].

## Material and methods

### Subjects

The sample of the study consisted of 6,631 handball players (3,358 men and 3,273 women) who had played for teams registered in the World Handball Championships, which are organised annually by the International Handball Federation (IHF). The IHF groups athletes according to their chronological age in categories of 2 years (in other words, in a biannual cycle), and uses January 1 as the start date for each two-year period. In the years 2013, 2015 and 2017, the World Handball Championships were for men and senior women; in 2014, 2016 and 2018, the Championships were for youth and junior women athletes. Players were allocated to one of three categories in the championships: youth or under 19 years old (U-19), junior or under 21 years old (U-21), and senior. Our study used the same allocation categories. However, in the youth and junior categories, players who were born outside the biannual cycle of international competition were excluded from the study because the sample could otherwise potentially have included duplicated subjects in the competition categories we analysed. Thus, 9.36% of all the male players (226) were excluded from the sample, and 9.35% of all the female players (229) were excluded. The athletes who were included were then further categorised according to their handball field positions, and by the continental federation to which their national team belonged. The sample of players was divided further by the year in which the World Championships took place (2013–14, 2015–16 or 2017–18) to prevent any players from being included again in subsequent analyses. This step was taken because some players participated in two different championship categories in the same year. The description and distribution of the handball players’ positions and continental federations, by competition and by category, are shown in Table 1.

**Table 1:** Description and distribution of male and female handball players’ position and continental federation, by competition and category.

			2013			2015			2017			TOTAL
			U-19[n(%)]	U-21[n(%)]	Sen[n(%)]	U-19[n(%)]	U-21[n(%)]	Sen[n(%)]	U-19[n(%)]	U-21[n(%)]	Sen[n(%)]	
M	Pos	Goalkeeper	52 (14%)	50 (14.5%)	55 (13.4%)	56 (15.2%)	46 (13.5%)	56 (13.7%)	52 (14.1%)	46 (13.5%)	58 (14.3%)	471
		Wing	92 (24.7%)	92 (26.7%)	99 (24.1%)	87 (23.6%)	81 (23.8%)	103 (25.2%)	92 (25.0%)	88 (25.9%)	97 (23.8%)	831
		Lateral Back	110 (29.6%)	108 (31.3%)	129 (31.5%)	112 (30.4%)	109 (32.1%)	120 (29.4%)	120 (32.6%)	104 (30.6%)	115 (28.3%)	1027
		Centre-Back	66 (17.7%)	49 (14.2%)	58 (14.1%)	56 (15.2%)	61 (17.9%)	59 (14.5%)	46 (12.5%)	52 (15.3%)	67 (16.5%)	514
		Pivot	52 (14.0%)	46 (13.3%)	69 (16.8%)	57 (15.5%)	43 (12.6%)	70 (17.2%)	58 (15.8%)	50 (14.7%)	70 (17.2%)	515
	Fed	CAHB	62 (16.7%)	71 (20.6%)	52 (12.7%)	45 (12.2%)	59 (17.4%)	51 (12.5%)	49 (13.3%)	75 (22.1%)	48 (11.8%)	512
		NACHC/SCAHC	57 (15.3%)	40 (11.6%)	52 (12.7%)	55 (14.9%)	66 (19.4%)	50 (12.3%)	50 (13.6%)	35 (10.3%)	50 (12.3%)	455
		AHF	48 (12.9%)	39 (11.3%)	53 (12.9%)	46 (12.5%)	49 (14.4%)	53 (13.0%)	45 (12.2%)	39 (11.5%)	70 (17.2%)	442
		EHF	205 (55.1%)	195 (56.5%)	237 (57.8%)	222 (60.3%)	166 (48.8%)	254 (62.3%)	224 (60.9%)	191 (56.2%)	239 (58.7%)	1933
		OCHF	0 (0%)	0 (0%)	16 (3.9%)	0 (%)	0 (%)	0 (%)	0 (%)	0 (%)	0 (%)	16
Male players by CAT [n (%)]			372 (33.0%)	345 (30.6%)	410 (36.4%)	368 (33.0%)	340 (30.5%)	408 (36.6%)	368 (33.0%)	340 (30.5%)	407 (36.5%)	3358
W	Pos	Goalkeeper	51 (14.5%)	52 (15.7%)	57 (14.5%)	54 (15.0%)	49 (14.2%)	58 (14.6%)	55 (14.9)	53 (16.2%)	55 (13.8%)	484
		Wing	92 (26.2%)	86 (25.9%)	91 (23.2%)	93 (25.8%)	90 (26.0%)	94 (23.7%)	89 (24.1%)	78 (23.8%)	89 (22.3%)	802
		Lateral Back	100 (28.5%)	90 (27.1%)	116 (29.6%)	111 (30.8%)	101 (29.2%)	127 (32.1%)	110 (29.8%)	90 (27.4%)	118 (29.6%)	963
		Centre-Back	57 (16.2%)	54 (16.3%)	68 (17.3%)	53 (14.7%)	58 (16.8%)	58 (14.6%)	61 (16.5%)	54 (16.5%)	71 (17.8%)	534
		Pivot	51 (14.5%)	50 (15.1%)	60 (15.3%)	49 (13.6%)	48 (13.9%)	59 (14.9%)	54 (14.6%)	53 (16.2%)	66 (16.5%)	490
	Fed	CAHB	40 (11.4%)	29 (8.7%)	81 (20.7%)	35 (9.7%)	45 (13.0%)	50 (12.6%)	41 (11.1%)	27 (8.2%)	48 (12.0%)	396
		NACHC/SCAHC	40 (11.4%)	42 (12.7%)	60 (15.3%)	62 (17.2%)	43 (12.4%)	64 (16.2%)	30 (8.1%)	38 (11.6%)	49 (12.3%)	428
		AHF	68 (19.4%)	56 (16.9%)	50 (12.8%)	68 (18.9%)	67 (19.4%)	64 (16.2%)	74 (20.1%)	42 (12.8%)	50 (12.5%)	539
		EHF	203 (57.8%)	205 (61.7%)	185 (47.2%)	195 (54.2%)	191 (55.2%)	218 (55.1%)	224 (60.7%)	221 (67.4%)	252 (63.2%)	1894
		OCHF	0 (0%)	0 (0%)	16 (4.1%)	0 (0%)	0 (0%)	0 (0%)	0 (0%)	0 (0%)	0 (0%)	16
Female players by CAT [n (%)]			351 (32.7%)	332 (30.9%)	392 (36.5%)	360 (32.7%)	346 (31.4%)	396 (35.9%)	369 (33.7%)	328 (29.9%)	399 (36.4%)	3273

U-19, youth category; U-21, junior category; Sen, senior category; M, men; W, women; Pos, playing position; Fed, national federation; CAT, category; CAHB, African Handball Confederation; NACHC, North America and the Caribbean Handball Confederation; SCAHC, South and Central America Handball Confederation; AHF, Asian Handball Federation; EHF, European Handball Federation; OCHF, Oceania Continent Handball Federation

### Procedures

All data were acquired from an IHF database and downloaded from the ´Competitions´ Section of the IHF website (https://www.ihf.info/competitions). Senior players’ birth dates were categorised into four quartiles because their selection year corresponded with the regular calendar year. However, the birth dates of the youth and junior players were divided into eight quarters, each corresponding with the eight quarters in the biannual time range. Players born in even numbered birth years were therefore included in one of the following groups: Quartile 1 (Q1)–players born between January 1 and March 31; Quartile 2 (Q2)–players born between April 1 and June 30; Quartile 3 (Q3)–players born between July 1 and September 30; Quartile 4 (Q4)–players born between October 1 and December 31. Players born in odd-numbered birth years were categorised as follows: Quartile 5 (Q5)–players born between January 1 and March 31; Quartile 6 (Q6)–players born between April 1 and June 30; Quartile 7 (Q7)–players born between July 1 and September 30; Quartile 8 (Q8)–players born between October 1 and December 31.

The sample of senior players was divided into two semesters. Further, youth players and junior players were assigned to one of four semesters in each category. Hence, for those athletes born in even-numbered birth years, the categories were as follows: Semester 1 (S1)–players born between January 1 and June 30; Semester 2 (S2)–players born between July 1 and December 31. For players born in odd-numbered birth years, the categories were as follows: Semester 3 (S3)–players born between January 1 and June 30; Semester 4 (S4)–players born between July 1 and December 31.

The following six continental federation categories were also included: the African Handball Confederation; the North America and the Caribbean Handball Confederation; the South and Central America Handball Confederation; the Asian Handball Federation; the European Handball Federation; and the Oceania Continent Handball Federation. The following age categories were analysed: U-19; U-21, and Senior. The following five playing positions were analysed: goalkeeper; wing; lateral back; center-back; and pivot. Table 1 shows the distribution of players by position, continental federation, gender and competition category.

Statistics about player performance were taken from the website of the IHF to correlate the following parameters with the potential impacts of RAE in handball: the *percentage of effectiveness in shot* (PESh), the *percentage of effectiveness in saves* (PESa), *assists*, *technical faults-turnovers* (TF-TO), *steals* (ST), *blocked shots* (BS), *penalties* (PEN), *minutes played* (MIN), and the championships ranking. The performance categories *steal* and *blocked shot* were not provided by the IHF for the youth and junior competitions. In the disputed Youth and Junior Women’s Championships of 2018, the category *time played* was not used. The number of handball penalties was quantified as the sum of the number of yellow cards *plus* the number of exclusions or red/blue cards. The classification of team performance was determined by the placement of each national team. In order to adjust for the individual performance of each player, all statistical data were normalised in relation to the number of minutes that each player played. Thus, the possible effect of time on each player was eliminated.

### Statistical analysis

Data analysis was conducted using the *Statistical Package for Social Sciences* (SPSS 23.0, IBM Corp., Armonk, NY, USA), and *Windows Office Excel 2010*. The differences between the observed and expected birth date distributions were tested using the chi-square goodness of fit test. In most countries, the distribution of births remains constant across the year, and therefore no significant variation was assumed. For the purposes of statistical analysis, the expected frequency of births in a quartile is 25%, assuming a homogeneous sample distribution [30].

We calculated odds ratios and 95% confidence intervals (CI) for the quartile and half-year distributions so that we could examine subgroup differences with respect to the potential non-uniformity of the birth date distribution. The odds ratios compared the birth date distribution of a particular quartile (Q1, Q2, Q3, Q4, Q5, Q6 or Q7) or semester (S1, S2 or S3) with a reference group consisting of the relatively youngest players (Q8 or S4). A higher odds ratio would indicate an increased likelihood of players who were born in that particular quartile compared to the reference quartile. In order to determine the strength of association, a Cramer’s V statistical test was applied, in which 0.10 to 0.20 indicated a weak association; 0.20 to 0.40, a moderate association; 0.40 to 0.60, a relatively strong association; 0.60 to 0.80, a strong association; and *Vc* > 0.80, a very strong association [31]. The level of significance was set at p<0.05. Data for players from the Oceania Continent Federation are only represented in Table 1. These players were excluded from the observed and expected birth date distributions analysis due to the small sample size (n = 32).

## Results

### Distribution by category

Table 2 shows the quarterly distribution of birth dates by category as a function of the competition level by age and the gender of the players. Separate chi-square analyses revealed that the birth date distribution by quartile differed significantly from the expected birth date distribution in the youth and junior categories (*p < 0*.*001*). The largest effect sizes observed were in the U-19 category for women in the 2014 and 2018 World Championships (*Vc = 0*.*48*), and in the U-19 category for men in the 2017 World Championship (*Vc = 0*.*52*). No significant differences were evident in the female senior players in the 2013, 2015 and 2017 World Championships, and the male senior players in the 2013 World Championship (*p > 0*.*05*). Similar findings were evident in the birth date distribution by semester (Table 3): significant values ​​were found in the male senior category in the three competitions analysed (2013 WC: *p < 0*.*05*; 2015 WC: *p < 0*.*01*: 2017 WC: *p < 0*.*05*). The largest effect size was found in the U-19 category for men in the 2017 World Championship (*Vc = 0*.*49*).

**Table 2:** Quarterly distribution (n (%) and odds ratio (OR)) of birth dates for the category subgroup as a function of the competition for male and female players.

		Q1		Q2		Q3		Q4		Q5		Q6		Q7		Q8		X^2^	gl	p	Vc
		n (%)	OR	n (%)	OR	n (%)	OR	n (%)	OR	n (%)	OR	n (%)	OR	n (%)	OR	n (%)	OR				
		2013–2014																			
M	U-19	86	0.8	72	0.8	51	0.6	42	0.7	36	0.8	34	1.5	29	0.6	22	-	73.63	7	<0.001	0.44
		(23.1%)		(19.4%)		(13.7%)		(11.3%)		(9.7%)		(9.1%)		(7.8%)		(5.9%)					
	U-21	65	0.9	43	0.6	66	1.3	40	0.9	45	0.9	32	0.6	30	1.1	24	-	38.88	7	<0.001	0.34
		(18.8%)		(12.5%)		(19.1%)		(11.6%)		(13.0%)		(9.3%)		(8.7%)		(7.0%)					
	Senior	108	0.9	118	1.0	97	0.8	87	-	-	-	-	-	-	-	-	-	5.29	3	0.153	0.11
		(26.3%)		(28.8%)		(23.7%)		(21.2%)													
W	U-19	74	1.2	68	1.3	64	1.7	43	1.4	33	1.3	17	0.7	36	1.7	16	-	81.48	7	<0.001	0.48
		(21.1%)		(19.4%)		(18.2%)		(12.3%)		(9.4)		(4.8%)		(10.3%)		(4.6%)					
	U-21	62	1.1	65	1.8	44	0.8	36	1.1	38	1.0	45	1.7	22	0.9	20	-	45.21	7	<0.001	0.37
		(18.7%)		(19.6%)		(13.3%)		(10.8%)		(11.4%)		(13.6%)		(6.6%)		(6.0%)					
	Senior	101	1.1	107	1.1	109	1.3	75	-	-	-	-	-	-	-	-	-	7.55	3	0.056	0.14
		(25.8%)		(27.3%)		(27.8%)		(19.1%)													
		2015–2016																			
M	U-19	66	1.2	76	2.4	62	2.1	45	1.6	51	2.4	34	1.8	19	1.1	15	-	74.26	7	<0.001	0.45
		(17.9%)		(20.7%)		(16.8%)		(12.2%)		(13.9%)		(9.2%)		(5.2%)		(4.1%)					
	U-21	65	1.6	58	1.7	46	1.7	49	2.6	44	1.9	25	1.5	37	1.8	16	-	43.34	7	<0.001	0.36
		(19.1%)		(17.1%)		(13.5%)		(14.4%)		(12.9%)		(7.4%)		(10.9%)		(4.7%)					
	Senior	120	1.2	118	1.2	88	0.8	82	-	-	-	-	-	-	-	-	-	11.53	3	0.009	0.17
		(29.4%)		(28.9%)		(21.6%)		(20.1%)													
W	U-19	89	0.8	52	0.4	49	0.5	47	0.6	36	0.4	32	0.6	30	0.9	25	-	64.00	7	<0.001	0.42
		(24.7%)		(14.4%)		(13.6%)		(13.1%)		(10.0%)		(8.9%)		(8.3%)		(6.9%)					
	U-21	70	0.6	61	0.6	48	0.6	33	0.4	40	0.5	30	0.7	36	0.6	28	-	37.68	7	<0.001	0.33
		(20.2%)		(17.6%)		(13.9%)		(9.5%)		(11.6%)		(8.7%)		(10.4%)		(8.1%)					
	Senior	103	0.8	103	0.9	107	1.2	83	-	-	-	-	-	-	-	-	-	3.56	3	0.314	0.09
		(26.0%)		(26.0%)		(27.0%)		(21.0%)													
		2017–2018																			
M	U-19	75	0.9	83	1.7	62	1.5	41	1.3	47	1.2	26	1.0	17	0.7	17	-	99.46	7	<0.001	0.52
		(20.4%)		(22.6%)		(16.8%)		(11.1%)		(12.8%)		(7.1%)		(4.6%)		(4.6%)					
	U-21	49	1.2	60	3.6	47	2.1	45	2.0	51	2.3	44	1.9	29	1.5	15	-	32.66	7	<0.001	0.31
		(14.4%)		(17.6%)		(13.8%)		(13.2%)		(15.0%)		(12.9%)		(8.5%)		(4.4%)					
	Senior	115	1.2	114	1.1	106	1.1	72	-	-	-	-	-	-	-	-	-	12.08	3	0.007	0.17
		(28.3%)		(28.0%)		(26.0%)		(17.7%)													
W	U-19	96	1.1	58	0.6	49	0.7	38	0.8	48	0.9	30	1.0	30	1.5	20	-	84.75	7	<0.001	0.48
		(26.0%)		(15.7%)		(13.3%)		(10.3%)		(13.0%)		(8.1%)		(8.1%)		(5.4%)					
	U-21	72	0.8	30	0.3	41	0.5	40	0.5	40	0.4	42	0.5	36	0.7	27	-	31.85	7	<0.001	0.31
		(22.0%)		(9.1%)		(12.5%)		(12.2%)		(12.2%)		(12.8%)		(11.0%)		(8.2%)					
	Senior	105	0.8	113	0.9	103	0.9	78	-	-	-	-	-	-	-	-	-	6.89	3	0.076	0.13
		(26.3%)		(28.3%)		(25.8%)		(19.5%)													

Q1-Q8, birth quarter; n (%), absolute-relative frequency; OR, odds ratio; X^2^, chi square; gl, degrees of freedom; p, level of significance, Vc, Cramer´s V; M, men; W, women; U-19, youth category; U-21, junior category

**Table 3:** Semester distribution (n (%) and odds ratio (OR)) of birth dates for the category subgroup as a function of the competition for male and female players.

		S1		S2		S3		S4		X^2^	gl	p	Vc
		n (%)	OR	n (%)	OR	n (%)	OR	n (%)	OR				
		2013–2014											
M	U-19	158	1.1	95	0.9	68	1.4	51	-	71.16	3	<0.001	0.44
		(42.5%)		(25.5%)		(18.3%)		(13.7%)					
	U-21	108	0.6	106	1.0	77	0.7	54	-	23.06	3	<0.001	0.26
		(31.3%)		(30.7%)		(22.3%)		(15.7%)					
	Senior	226	1.1	184	-	-	-	-	-	4.30	1	0.038	0.10
		(55.1%)		(44.9%)									
W	U-19	142	0.8	107	1.1	50	0.7	52	-	68.57	3	<0.001	0.44
		(40.5%)		(30.5%)		(14.2%)		(14.8%)					
	U-21	127	1.5	80	1.0	84	1.4	41	-	44.70	3	<0.001	0.37
		(38.3%)		(24.1%)		(25.3%)		(12.3%)					
	Senior	208	0.9	184	-	-	-	-	-	1.47	1	0.225	0.06
		(53.1%)		(46.9%)									
		2015–2016											
M	U-19	142	1.6	107	1.8	85	2.0	34	-	66.72	3	<0.001	0.43
		(38.6%)		(29.1%)		(23.1%)		(9.2%)					
	U-21	124	1.1	94	1.4	69	1.2	53	-	33.91	3	<0.001	0.32
		(36.5%)		(27.6%)		(20.3%)		(15.6%)					
	Senior	238	1.3	170	-	-	-	-	-	11.33	1	0.001	0.17
		(58.3%)		(41.7%)									
W	U-19	140	0.6	97	0.6	68	0.5	55	-	47.31	3	<0.001	0.36
		(38.9%)		(26.9%)		(18.9%)		(15.3%)					
	U-21	131	0.9	81	0.7	70	0.8	64	-	32.24	3	<0.001	0.31
		(37.9%)		(23.4%)		(20.2%)		(18.5%)					
	Senior	206	0.8	190	-	-	-	-	-	0.65	1	0.421	0.04
		(52.0%)		(48.0%)									
		2017–2018											
M	U-19	157	1.5	104	1.8	74	1.4	33	-	88.85	3	<0.001	0.49
		(42.7%)		(28.3%)		(20.1%)		(9.0%)					
	U-21	110	1.5	91	1.6	95	1.7	44	-	28.73	3	<0.001	0.29
		(32.4%)		(26.8%)		(27.9%)		(12.9%)					
	Senior	229	1.1	178	-	-	-	-	-	6.39	1	0.011	0.12
		(56.3)		(43.7%)									
W	U-19	154	0.7	87	0.6	79	0.7	49	-	63.81	3	<0.001	0.42
		(41.7%)		(23.6%)		(21.4%)		(13.3%)					
	U-21	102	0.6	81	0.6	82	0.6	63	-	9.29	3	0.026	0.17
		(31.1%)		(24.7%)		(25.0%)		(19.2%)					
	Senior	219	0.9	180	-	-	-	-	-	3.81	1	0.051	0.10
		(54.9%)		(45.1%)									

S1-S4, birth semester; n (%), absolute-relative frequency; OR, odds ratio; X^2^, chi square; gl, degrees of freedom; p, level of significance; Vc, Cramer´s V; M, men; W, women; U-19, youth category; U-21, junior category

### Distribution by position

Table 4 shows the quarterly distribution of the birth dates by player position as a function of the competition period and the gender of the players. Separate chi-square analyses revealed that the birth date distributions by quartile differed significantly from the observed birth date distribution in the five player positions analysed (*p < 0*.*001*). The largest effect size we found was for women playing as center backs at the women’s World Championships of 2013/2014 (*Vc = 0*.*71*), 2015–2016 (*Vc = 0*.*65*) and 2017/2018 (*Vc = 0*.*71*). For men, the largest effect size was among those playing in the position of pivot (2013–2014 WC: *Vc = 0*.*66*; 2015–2016 WC: *Vc = 0*.*68*; 2017–2018 WC: *Vc = 0*.*60*). Similar results were observed in the birth date distribution by semesters (*p < 0*.*001*) (Table 5). The largest effect size was for women in the position of center back (2013–2014 WC: *Vc = 0*.*68*; 2015–2016 WC: *Vc = 0*.*61*; 2017–2018 WC: *Vc = 0*.*62*), and for men in the pivot position (2013–2014 WC: *Vc = 0*.*66*; 2015–2016 WC: *Vc = 0*.*67*; 2017–2018 WC: *Vc = 0*.*61*).

**Table 4:** Quarterly distribution (% and odds ratio (OR)) of birth dates for the position subgroup as a function of the competition by gender.

		Q1		Q2		Q3		Q4		Q5		Q6		Q7		Q8		X^2^	gl	p	Vc
		%	OR	%	OR	%	OR	%	OR	%	OR	%	OR	%	OR	%	OR				
		2013–2014																			
M	GK	18.5	0.63	20.4	0.80	16.6	0.61	15.3	0.75	7.0	1.07	10.2	1.04	6.4	0.98	5.7	-	30.26	7	<0.001	0.44
	W	21.6	0.64	20.5	0.70	22.3	0.76	14.1	0.62	7.1	0.49	5.3	0.51	4.6	0.47	4.6	-	101.93	7	<0.001	0.60
	LB	24.5	0.99	21.6	0.65	18.7	0.63	15.0	0.77	8.1	0.93	4.3	0.67	5.2	0.71	2.6	-	141.60	7	<0.001	0.64
	CB	23.1	1.18	17.3	1.02	19.7	1.44	15.6	1.64	6.9	1.40	7.5	1.66	6.9	4.20	2.9	-	52.06	7	<0.001	0.55
	P	26.3	0.95	22.8	0.76	15.6	0.67	15.6	0.99	6.0	1.33	4.2	0.93	3.6	0.40	6.0	-	73.34	7	<0.001	0.66
W	GK	22.5	1.60	19.4	1.25	20.6	1.63	15.6	1.34	5.0	0.94	7.5	0.96	5.0	1.03	4.4	-	54.60	7	<0.001	0.58
	W	21.9	1.57	19.0	1.43	19.0	1.32	14.9	1.63	9.3	2.03	6.7	1.95	6.3	2.13	3.0	-	75.54	7	<0.001	0.53
	LB	18.6	1.01	25.2	1.54	22.5	1.59	14.7	1.30	6.5	1.07	4.9	1.50	5.6	1.42	2.0	-	136.20	7	<0.001	0.63
	CB	26.8	0.86	22.9	0.98	18.4	0.69	12.8	0.61	6.7	0.71	6.1	0.60	2.2	0.24	3.9	-	86.16	7	<0.001	0.71
	P	23.0	1.05	24.8	1.32	19.3	1.49	13.0	1.01	3.7	0.75	3.7	1.07	7.5	2.50	5.0	-	70.11	7	<0.001	0.65
		2015–2016																			
M	GK	25.9	2.70	20.3	2.76	19.0	2.42	13.9	2.62	10.8	3.27	5.1	2.0	3.8	1.07	1.3	-	68.94	7	<0.001	0.66
	W	14.8	1.43	24.0	2.63	20.7	2.14	17.0	2.05	10.7	4.14	5.2	2.00	5.2	2.00	2.6	-	93.84	7	<0.001	0.59
	LB	25.8	2.32	22.3	2.20	14.7	1.59	15.2	1.96	9.1	2.39	4.7	1.78	5.6	2.38	2.6	-	137.19	7	<0.001	0.40
	CB	22.7	0.48	22.2	0.62	17.6	0.52	15.9	0.97	3.4	0.26	6.8	0.61	6.3	0.61	5.1	-	62.55	7	<0.001	0.36
	P	24.7	2.50	23.5	3.33	17.1	3.45	16.5	2.80	7.1	3.33	5.3	2.81	3.5	1.36	2.4	-	77.81	7	<0.001	0.68
W	GK	23.6	0.37	18.0	0.36	19.3	0.41	13.0	0.38	8.1	0.31	6.2	0.50	8.7	0.93	3.1	-	46.55	7	<0.001	0.54
	W	21.7	0.70	19.1	0.38	20.2	0.47	17.3	0.49	5.4	0.24	5.4	0.50	5.4	0.50	5.4	-	91.20	7	<0.001	0.57
	LB	22.4	0.43	20.4	0.45	18.6	0.63	15.6	0.51	7.7	0.42	5.3	0.56	4.7	0.42	5.3	-	106.90	7	<0.001	0.56
	CB	27.2	2.07	20.7	1.62	19.5	1.92	9.5	1.03	7.7	3.90	6.5	1.65	5.9	1.64	3.0	-	72.47	7	<0.001	0.65
	P	26.9	0.40	19.2	0.30	13.5	0.29	16.0	0.36	5.8	0.30	5.1	0.36	7.1	0.73	6.4	-	54.05	7	<0.001	0.59
		2017–2018																			
M	GK	23.7	2.17	21.2	3.96	21.8	4.25	12.2	2.38	9.6	3.75	4.5	2.10	5.1	3.00	1.9	-	65.64	7	<0.001	0.65
	W	17.7	1.72	20.9	2.32	22.4	2.70	16.2	2.25	10.5	2.52	6.1	2.13	4.0	1.83	2.2	-	96.17	7	<0.001	0.59
	LB	23.6	1.67	24.2	2.05	19.2	1.95	11.5	1.36	7.7	1.35	5.6	1.36	4.7	0.96	3.5	-	140.22	7	<0.001	0.64
	CB	19.4	0.96	26.1	2.08	16.4	0.86	15.2	1.79	9.1	1.50	9.1	2.50	3.6	0.90	1.2	-	63.70	7	<0.001	0.62
	P	23.0	0.53	23.0	0.65	15.2	0.58	16.9	0.60	7.3	0.80	6.7	0.42	2.8	0.25	5.1	-	65.15	7	<0.001	0.60
W	GK	31.3	0.46	15.3	0.25	14.7	0.24	14.7	0.42	7.4	0.27	6.1	0.48	4.9	0.33	5.5	-	70.96	7	<0.001	0.66
	W	22.3	0.58	19.5	0.43	18.0	0.37	15.6	0.44	9.0	0.40	6.3	0.47	4.7	0.55	4.7	-	73.31	7	<0.001	0.53
	LB	22.6	0.60	18.9	0.49	15.7	0.51	13.5	0.74	9.1	0.74	6.6	0.74	7.9	1.04	5.7	-	68.52	7	<0.001	0.46
	CB	26.9	1.04	16.7	0.49	25.3	1.16	11.3	0.56	8.1	0.67	4.8	0.40	5.4	1.11	1.6	-	94.69	7	<0.001	0.71
	P	24.9	1.89	20.2	1.54	15.0	1.73	16.2	1.68	5.2	1.25	9.2	2.40	6.4	3.96	2.9	-	59.00	7	<0.001	0.58

Q1-Q8, birth quarter; (%), relative frequency; OR, odds ratio; X^2^, chi square; gl, degrees of freedom; p, level of significance; Vc, Cramer´s V; M, men; W, women; GK, goalkeeper; W, wing; LB, lateral back; CB, centre back; P, pivot

**Table 5:** Semester distribution (% and odds ratio (OR)) of birth dates for the position subgroup as a function of the competition for male and female players.

		S1		S2		S3		S4		X^2^	gl	p	Vc
		%	OR	%	OR	%	OR	%	OR				
		2013–2014											
M	GK	38.9	0.68	31.8	0.65	16.6	0.98	12.7	-	28.91	3	<0.001	0.43
	W	42.0	1.00	36.7	1.06	12.0	0.71	9.2	-	95.93	3	<0.001	0.58
	LB	46.1	1.05	34.0	0.92	12.4	1.09	7.5	-	137.72	3	<0.001	0.63
	CB	40.5	0.51	35.3	0.71	14.5	0.70	9.8	-	47.46	3	<0.001	0.52
	P	49.1	1.35	31.1	1.23	10.2	1.78	9.6	-	71.87	3	<0.001	0.66
W	GK	41.9	1.46	36.3	1.55	12.5	1.03	9.4	-	51.95	3	<0.001	0.57
	W	40.9	1.00	33.8	0.95	16.4	1.40	8.9	-	71.42	3	<0.001	0.51
	LB	44.1	0.95	36.9	1.08	11.4	0.92	7.5	-	122.08	3	<0.001	0.63
	CB	49.7	1.97	31.3	1.42	12.8	1.42	6.1	-	82.61	3	<0.001	0.68
	P	47.2	0.74	32.9	0.82	7.5	0.57	12.4	-	65.81	3	<0.001	0.64
		2015–2016											
M	GK	46.2	2.59	32.9	2.38	15.8	2.58	5.1	-	62.81	3	<0.001	0.63
	W	38.7	1.33	37.6	1.40	15.9	2.05	7.7	-	79.10	3	<0.001	0.54
	LB	48.4	1.39	29.6	1.05	13.8	1.30	8.2	-	133.12	3	<0.001	0.62
	CB	44.9	0.73	33.5	0.90	10.2	0.56	11.4	-	61.41	3	<0.001	0.59
	P	48.2	2.39	33.5	2.60	12.4	2.59	5.9	-	77.39	3	<0.001	0.67
W	GK	41.6	0.38	32.3	0.42	14.3	0.39	11.8	-	39.82	3	<0.001	0.50
	W	40.8	0.75	37.5	0.71	10.8	0.49	10.8	-	89.57	3	<0.001	0.57
	LB	42.5	0.72	34.5	0.95	13.0	0.77	10.0	-	103.68	3	<0.001	0.55
	CB	47.9	1.37	29.0	1.11	14.2	1.78	8.9	-	62.08	3	<0.001	0.61
	P	46.2	0.42	29.5	0.38	10.9	0.39	13.5	-	49.90	3	<0.001	0.57
		2017–2018											
M	GK	44.9	1.42	34.0	1.71	14.1	1.55	7.1	-	57.18	3	<0.001	0.61
	W	38.6	1.34	38.6	1.70	16.6	1.56	6.1	-	88.39	3	<0.001	0.56
	LB	47.5	1.87	31.0	1.73	13.3	1.38	8.3	-	130.09	3	<0.001	0.62
	CB	45.5	1.51	31.5	1.24	18.2	2.03	4.8	-	60.29	3	<0.001	0.60
	P	46.6	1.31	31.5	1.28	14.6	1.28	7.3	-	66.27	3	<0.001	0.61
W	GK	46.6	0.70	29.4	0.59	13.5	0.65	10.4	-	54.25	3	<0.001	0.58
	W	42.2	0.75	33.2	0.59	15.6	0.64	9.0	-	72.41	3	<0.001	0.53
	LB	41.5	0.53	29.2	0.58	15.7	0.72	13.5	-	64.67	3	<0.001	0.45
	CB	43.5	0.67	36.6	0.81	12.9	0.49	7.0	-	70.56	3	<0.001	0.62
	P	45.1	0.76	31.2	0.78	14.5	0.78	9.2	-	55.46	3	<0.001	0.57

S1-S4, birth semester; (%), relative frequency; OR, odds ratio; X^2^, chi square; gl, degrees of freedom; p, level of significance, Vc, Cramer´s V; M, men; W, women; GK, goalkeeper; W, wing; LB, lateral back; CB, centre back; P, pivot

Tables 2, 3, 4 and 5 show the odds ratio calculations. This category and position analysis by quartile and semester revealed no significant odds ratios when the observed and the expected birth date distributions were compared.

### Distribution by continental federation

**Fig 1:**
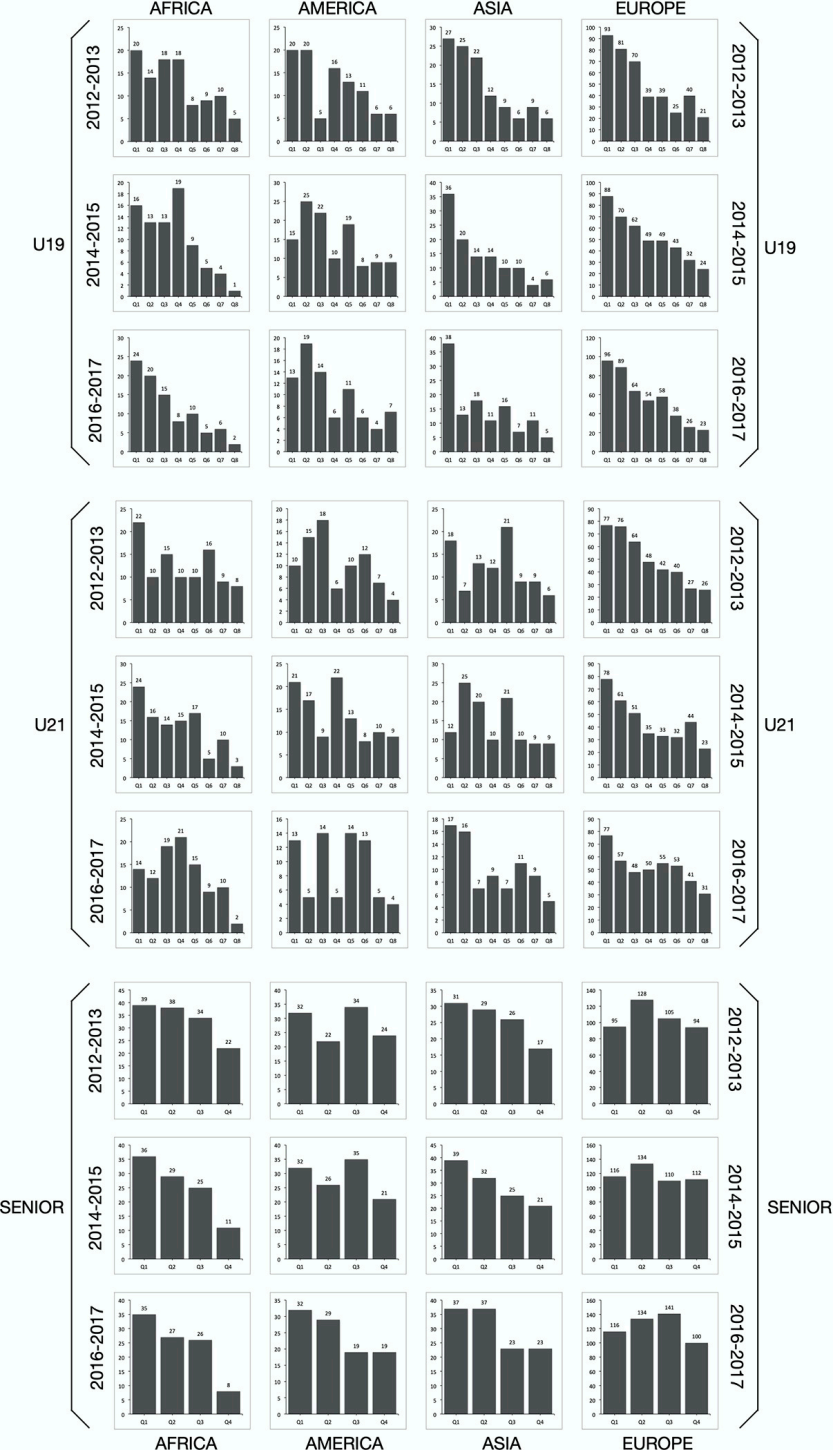
Quarterly distribution (relative [%] and absolute frequency) of birth dates for the continental federation subgroup as a function of the competition and category.

In addition, we performed a chi-square analysis to investigate the relationship between the relative age of the players in the continental federations (Fig 1). Significant differences in each of the confederations were found (*p<0*.*05*) in the U-19 and U-21 categories. No significant findings were found in the senior category (*p>0*.*05*), except among athletes in the African Handball Federation (*p<0*.*01)* in the 2015/16 and 2017/18 World Championships, and for the European Handball Federation (*p<0*.*05*) in the 2017/18 World Championships.

### Correlations between relative age and statistics on individual performance

Positive correlations were observed between the relative age of the players and the number of minutes played (*p<0*.*05*) for both genders, except in the female senior category (*p>0*.*05*) (Table 6). The findings were especially significant in the youth category (*p<0*.*01*). This means that relatively older athletes played more minutes than younger athletes in their corresponding competition categories. Relative age was correlated significantly (*p<0*.*01*) to the number of assists in the youth and junior female categories, and penalties in youth and junior male and youth female categories. Relative age correlated positively (*p<0*.*05*) to the percentage of shot effectiveness in the male senior category, technical faults-turnovers in the male and female youth and male junior categories, and the number of steals in the male senior category.

**Table 6:** Correlations between relative age and performance statistics by categories for male and female handball players.

Relative age effect						
	Men			Women		
	U-19	U-21	Senior	U-19	U-21	Senior
Min	-0.080**	-0.061*	0.060*	-0.104**	-0.083*	-0.009
PESh	0.020	-0.014	0.053*	-0.002	-0.008	0.032
PESa	-0.061	-0.055	0.099	0.012	-0.098	-0.013
AS	-0.013	-0.049	0.032	-0.095**	-0.095**	0.012
TF-TO	-0.052*	-0.073*	0.009	-0.055*	-0.038	-0.024
ST	-	-	0.049*	-	-	0.006
BS	-	-	0.042	-	-	-0.052
PEN	-0.075**	-0.102**	0.003	-0.084**	-0.042	-0.027
Classification	0.013	0.072*	-0.115**	0.066*	0.089**	-0.064*

U-19, youth category; U-21, junior category; Min, minutes played; PESh, percentage of effectiveness in shots; PESa, percentage of effectiveness in saves; AS, assists; TF-TO, technical faults-turnovers; ST, steals; BS, blocked shots; PEN, penalties. Significance value

*p<0.05

**p<0.01

***p<0.005

Table 7 shows the correlations between relative age and individual performance statistics per minute played. We found that relative age was correlated positively (*p<0*.*05*) with the percentage of effectiveness in saves per minute in the male senior category and female youth category, assists per minute in the female junior category, and penalties per minute in the male junior category. Significant positive correlations (*p<0*.*01*) were found in the percentage of effectiveness in shots in the male youth category, and the percentage of effectiveness in saves per minute in the male senior category.

**Table 7:** Correlations between relative age and performance statistics by categories for male and female handball players, per played time (min).

Relative age effect						
	Men			Women		
	U-19	U-21	Senior	U-19	U-21	Senior
PESh/min	0.092**	0.049	0.017	0.026	-0.022	0.021
PESa/min	-0.026	-0.037	-0.236**	0.153*	0.050	0.021
AS/min	0.015	-0.029	0.024	-0.030	-0.062*	0.030
TF-TO/min	0.006	-0.032	-0.014	-0.017	-0.020	-0.008
ST/min	-	-	0.025	-	-	0.007
BS/min	-	-	0.034	-	-	-0.025
PEN/min	-0.039	-0.068*	-0.015	-0.010	-0.004	-0.023

U-19, youth category; U-21, junior category; PESh/min, percentage of effectiveness in shots/minutes; PESa/min, percentage of effectiveness in saves/minutes; AS/min, assists/minutes; TF-TO/min, technical faults-turnovers/minutes; ST/min, steals/minutes; BS/min, blocked shots/minutes; PEN/min, penalties/minutes. Significance value

*p<0.05

**p<0.01

***p<0.005

### Correlations between relative age and final classification

Significant correlations were found between relative age and the final placement classification of each national team in the respective World Championships in all categories, except in the male youth category (*p>0*.*05*) (Table 6). This indicated that there was an over-representation of relatively older players in the national teams that reached the top positions in the World Handball Championships.

## Discussion

The key purpose of this study was to examine the RAE in the context of the highest levels of international handball competition. Our research focussed specifically on whether age affects the availability of opportunities in the sporting careers of male and female athletes. As expected, we observed a skewed distribution of birth dates in the youth and junior categories and noted that relatively older players were overrepresented. At the senior level, no uneven birth date distribution was observed among female players in any of the Championships. Non-uniform birth date distributions were observed for male players in the Championships of 2015 and 2017. The observed tendency of a decrease in the RAE at the senior level mirrors similar findings from other studies of team sports [32] and individual disciplines [33,34]. This suggests that the advantages athletes gain due to their early maturational development gradually decrease as they transition towards professional sport [2,35]. In addition, our study showed that the RAE was prevalent throughout the entire period analysed (2013–2018), both for male and female players.

The persistence of the RAE in handball is far from surprising due to the high correlation that has been observed between the level of athlete performance and the physical and physiological demands that are made [36,37]. Anthropometric parameters, such as weight, body mass, height, and arm length and leg length, strongly influence performance in youth handball [38]. It would be reasonable to suggest, therefore, that relatively older players should be expected to score better in these measurements compared to younger players during adolescence, and that they would be more likely to achieve better high-performance success. Because these capacities tend to be equated with physical maturity, the impacts of the RAE in senior handball categories therefore decrease or disappear altogether when athletes reach the senior categories, especially for women [39].

In the analysis of the relationship between the date of birth and the gender of athletes, the RAE was found to impact on the categories and competitions we analysed, except among female athletes in the senior World Championships. Our findings stand in contrast to other studies that reported no RAE impacts on athletes taking part in women´s sport, including in handball and other sports [40–42]. However, these other studies were based on samples of female players who belonged to teams participating in club competitions and may therefore be of less relevance to our findings because they do not compare similar impacts in terms of level of professionalism.

Studies that have investigated athlete selection to international or national youth and junior teams within TID systems in handball have reported similar RAE impacts in women’s handball [27,43,44]. The fact that the RAE was not found to have an impact on athletes in the senior category for women can be explained by the depth of competition in women’s handball and other sociocultural factors across and within female sporting settings [20].

Our results confirm findings from previous research that has shown that the influence of the RAE decreases as the level of competition increases [45,46]. This relationship, however, is not always consistent [47]. Studies of the RAE in systems of talent identification and development have shown, for example, how relatively younger players in Rugby Union games are less likely to be selected, but more likely to achieve senior professional status [48,49]. McCarthy et al. [50] have suggested that this may be due to psychological reasons. As players progress towards the senior categories, the researchers suggest, the additional, demanding challenges experienced by relatively younger players may be beneficial to their development by exposing them to more adverse developmental experiences. This may help the players to learn how to cope better with setbacks, uncertainties, and challenges. Other possible explanations may be that relatively younger athletes need to develop specific technical and/or tactical skills to reach high levels of performance despite their later maturation [51], and that the influence of other secondary factors (genetics, family, coaches, injuries, opportunities) can also help to turn later developers into experts [52]. The initial differences in the size and the physical performance between relatively younger athletes and their peers at selection would typically be of importance at the time when athletes transition to senior sports. Thus, some studies have indicated that relatively younger athletes, if selected, can perform better later [47], be injured less [53] and earn more [54] at the senior level, compared to their relatively older peers.

In the analysis of the relationship between the dates of birth of the athletes and playing positions, the RAE appears to be one of the factors that determine athlete selection for particular field positions [39,55]. Our findings showed that the RAE affected athlete selection for all playing positions in handball, especially for male pivots and goalkeepers, and for female center backs. As hypothesised, the RAE was evident among female back players. Anthropometric and physical requirements in handball, such as the need to have a large and strong body, high strength levels, and a high throwing velocity [56–58], mean that relatively older players tend to be in back positions, compared to relatively young players [59]. Unlike previous studies of male handball players that also included an analysis of playing positions [28,39], we found a higher prevalence of the RAE on the selection of athletes to the positions of pivots and goalkeepers. Large anthropometric size and strong physical capacities are required to compete in elite handball in general, and in goalkeeper and pivot positions in particular [60], but it seems that position-specific skill demands may also explain the RAE on these two playing positions [61]. It seems logical, we suggest, to think that relatively older players, who have enjoyed more and better training experiences (more skilled coaches, higher competition levels, better facilities and sport programmes) than their relatively younger counterparts [62], therefore have an advantage when selections are made for these positions.

In the analysis of the relationship between the athletes´ dates of birth and the differences in their continental federations, we observed that the RAE impacted athletes across all geographical settings, except Australia (the only participating country in the Oceania Confederation). One possible explanation for this is that the handball cut-off date used by countries such as Australia is August 1, instead of the more common, and internationally recognised date of January 1.

In our analysis of the relationship between the athletes´ dates of birth and the performance parameters, we observed that relatively older players, except those in the female senior category, played more minutes compared to relatively younger players. Consequently, older participating players obtained better scores on some performance parameters, such as technical faults-turnovers, assists and penalties in the youth and junior categories. In the male senior category, older athletes achieved higher effectiveness percentages in shots and steals. This finding is contrary to the results reported by Bjørndal et al. [27] who observed that the RAE did not impact the number of matches that athletes played in after they were initially selected to a team. Surprisingly, these study findings indicated that while international team selections favoured relatively older players, they did not strongly affect the possibility of players achieving a successful international career after first being selected. Although their study examined the number of match appearances, and our analysis in this study has focused on playing time, the contrasting results may indicate differences in the selection mechanisms used in other national systems of talent development. For example, some systems may be based on a broader population of players; and/or may have players who enter at only one stage (as opposed to different stages) throughout the lifetime of an international team. Collectively, however, the results clearly indicate that the maturational development of athletes does influence some individual performance parameters, especially the number of minutes played by athletes in the youth categories (U-19 and U-21). This impact continues, though to varying degrees, beyond the initial selection stages in different countries [12,28,63].

By gender, differences were observed regarding the individual player performance. Relatively older female handball players (in the U-19 and U-21 categories) produced more assists, both in absolute numbers and relative to the amount of playing time. This may possibly also be due to anthropometric parameters, such as hand length. Older players, as we have suggested, are more likely to have larger hand sizes. This would give them a more stable ball grip and, therefore, better pass action techniques [64,65]. However, given that players in this category are already in the later stages of adolescence, their biological maturation would typically either be near completion or finished. A more plausible reason is therefore likely to be that relatively older female players have more high-level competitive experience, and therefore have better decision-making skills compared to relatively younger players [66].

The total number of technical faults and turnovers was found to be higher for relatively older male players (in the U-19 and U-21 categories). While this could be seen as a sign of a decline in the individual performance of athletes, this difference disappears when this parameter is weighted by the number of minutes played. Potential explanations for this finding may be that relative older players play more minutes and are therefore likely to make more mistakes. However, because they are more experienced than their younger counterparts, they might also be assumed to make fewer mistakes per minute played. A similar age variation was found with regard to penalties.

Interestingly, the analysis of the relationship between date of birth and team performance showed that the RAE was associated with the final placement in the Championships, except for players in the male youth category. This finding is contrary to the results reported in other studies. Werneck et al. [67], for example, found no relationship between relative age and team performance in their sample of senior basketball players participating in the London Olympic Games in 2012. Similarly, Kirkendall [68] found no link between the RAE and team performance among U-16 players in North American football clubs. We assume that our findings differ with respect to the aforementioned studies for two reasons: (a) in the first study, the sample only included senior players, who had not taken part in the youth and junior categories; (b) in the second study, the analysis focused on a regular competitive club season. In comparison, the competition structure of the World Handball Championships does not necessarily reward teams that win the most matches.

Our findings suggest that high-performance handball competitions are biased in favour of relatively older players, and that the selection-based models currently in use may conflate the future potential of athletes with their current performance. Relatively older players typically belong to teams that achieve the best final placements in competitions and benefit from high quality training conditions (such as better sports facilities, more qualified coaches, and higher competition levels). This is more likely to enable players to achieve higher performance levels [62,63]. Hancock et al. [69] have argued that the RAE in sports can be explained largely by the self-reinforcing effects of psychological expectations that spur parents, coaches and athletes to respond in particular ways. For example, Cobley et al. [70] demonstrated how relatively younger players were less likely to participate in Rugby Union games from the U-13 to U-19 level. This necessarily affects both their initial selection and the later self-reinforcing mechanisms that create and sustain unequal opportunities for developing athletes [71]. It may also contribute to increases in drop-out rates among relatively young players in national TID systems [46]. It is important therefore to reflect on whether youth international competitions favour or harm participation and development in youth sport, and in youth handball in particular [72].

This study has also shown how the RAE relates to individual and team performance. It suggests that there are strong arguments to be made against selection-based models that appear to conflate future potential with current performance. The introduction of international championships for youth may only increase the risk of athletes being given unequal opportunities. This should be a great concern to policy makers and practitioners alike, as the effects of unequal opportunities is detrimental to the values of most sport organisations and may also be counterproductive to talent development, in particular.

It is important that equal opportunities are available to all players. Our study findings indicate that the time may be right to question the value and appropriateness of the TID systems currently being used in national and international handball, including the introduction of international championships for youth athletes. Particular attention needs to be given to athletes who are less likely to enjoy the benefits of early maturational development.

The physiological, kinanthropometric, conditional and psychological profile of players should be understood as a form, or framework, for guiding the talent development of athletes, but not as a selection tool [73]. Thus, one possible strategy to reduce the impacts of the RAE in TID systems could be to also include psychosocial factors in the existing selection tests. Variables related to training, leadership, and as cognitive competencies [74] could be assessed, being carried out under competition conditions. These could including not only gestures and decision-making, but also fatigue and other external conditions [75]. A key indicator of determining the impact of the RAE could be how well athletes learn in training and competition situations [76]–understood as the speed at which athletes acquire new skills or improve in ability. Other possible strategies to reduce the impacts of the RAE include making coaches more actively aware of how the RAE impacts selection processes [27] and creating more opportunities for athletes who are relatively younger. Adjusting the age categories used in youth and junior international competitions, so that the older players in a group could change category each year [44], could also be a way to improve current systems.

Our study had some limitations. Firstly, we did not know the distribution of birth dates among the wider populations of the countries analysed [39]. Secondly, the interpretations of the RAE we have presented here are potentially limited by the fact that a biased distribution may already be apparent in the population of licensed players. An asymmetry in birth date distributions would be expected to occur at all selection levels [46]. Further investigations of athlete selection to TID systems should attempt to investigate such concerns. Third, the absence of a consensus-based global individual performance index makes comparisons of the impacts of the RAE and individual performance difficult. Finally, our design did not allow us to address questions of causality.

## Conclusions

This is the first large-scale study of international handball to demonstrate that the RAE has a significant effect on male and female athlete selection to international handball competitions in the U-19, U-21, and (only in men) the senior categories. In addition, it is one of the first studies to demonstrate that the RAE in international handball remains constant over time. The study has shown further how the RAE relates to other variables, such as athletes´ playing positions, continental federation membership, and individual and team performance. The study contributes to the growing body of evidence showing that the impacts of the RAE are persistent and affect athletes across the world.

Future research should continue to focus on reviews of national talent development and identification systems in handball, including the selection processes associated with international youth team activities. This will enable a deeper exploration of how organisational differences and sociocultural factors influence the initial selections of players and, subsequently, their retention. Future research designs should allow for comparisons between countries and contexts. An examination of *when* selection mechanisms and the RAE begin to influence opportunities for the participation, development and performance of children and youth in different sport contexts. More interpretive research may also help researchers to move beyond descriptive overviews of the impacts of the RAE in sports and help to develop and reveal more appropriate strategies for talent identification and development [77].

## Supporting information

S1 Data Data from players participants in the 2013 World Championship. (XLSX)

S2 Data Data from players participants in the 2015 World Championship. (XLSX)

S3 Data Data from players participants in the 2017 World Championship. (XLSX)

S1 Fig Quarterly distribution (relative [%] and absolute frequency) of birth dates for the continental federation subgroup as a function of the category and the competition. (TIFF)

## References

[1] De Bosscher V, De Knop P, Van Bottenburg M, Shibli S. A Conceptual Framework for Analysing Sports Policy Factors Leading to International Sporting Success. European Sport Management Quarterly. 2006;6(2):185–215.

[2] Bjorndal CT, Luteberget LS, Holm S. The Relationship Between Early and Senior Level Participation in International Women ‘ s and Men ‘ s Handball by. Journal of Human Kinetics. 2018;63(August):73–84. 30279943 10.2478/hukin-2018-0008 PMC6162979

[3] Singer RN, Janelle CM. Determining sport expertise: from genes to supremes. International Journal of Sport Psychology. 1999;30(2):117–50.

[4] Bailey R, Collins D. The Standard Model of Talent Development and Its Discontents. Kinesiology Review. 2013;2(4):248–59.

[5] Helsen WF, Van Winckel J, Williams AM. The relative age effect in youth soccer across Europe. Journal of Sports Sciences. 2005;23(6):629–36. 10.1080/02640410400021310 16195011

[6] Johnston K, Wattie N, Schorer J, Baker J. Talent Identification in Sport: A Systematic Review. Sports Medicine. 2017;48(1):97–109. 10.1007/s40279-017-0803-2 29082463

[7] Torres-Unda J, Zarrazquin I, Gil J, Ruiz F, Irazusta A, Kortajarena M, et al Anthropometric, physiological and maturational characteristics in selected elite and non-elite male adolescent basketball players. Journal of Sports Sciences. 2013;31(2):196–203. 10.1080/02640414.2012.725133 23046359

[8] Turnnidge J, Hancock DJ, Côté J. The influence of birth date and place of development on youth sport participation. Scandinavian Journal of Medicine and Science in Sports. 2014;24(2):461–8. 10.1111/sms.12002 22998526

[9] Cobley SP, Baker J, Wattie N, McKenna J. Annual age-grouping and athlete development. A meta-analytical review of relative age effects in sport. Sports Medicine [Internet]. 2009;39(3):235–56. Available from: http://www.embase.com/search/results?subaction=viewrecord&from=export&id=L354314323%5Cn 10.2165/00007256-200939030-00005 19290678

[10] Deaner RO, Lowen A, Cobley S. Born at the wrong time: Selection bias in the NHL Draft. PLoS ONE. 2013;8(2):1–7. 10.1371/journal.pone.0057753 PMC3584041 23460902

[11] López de Subijana C, Lorenzo J. Relative Age Effect and Long-Term Success in the Spanish Soccer and Basketball National Teams. Journal of Human Kinetics. 2019;65(1):197–204. 10.2478/hukin-2018-0027 PMC6341957 30687431

[12] Ibañez SJ, Mazo A, Nascimento J, Garcıa-Rubio J. The relative age effect in under-18 basketball: Effects on performance according to playing position. PLoS ONE. 2018;13(7):1–11. 10.1371/journal.pone.0200408 PMC6037434 29985940

[13] Romann M, Fuchslocher J. Influences of player nationality, playing position, and height on relative age effects at women’s under-17 FIFA World Cup. Journal of Sports Sciences. 2013;31(1):32–40. 10.1080/02640414.2012.718442 22909307

[14] Lupo C, Boccia G, Ungureanu A, Frati R, Marocco R, Brustio PR. The beginning of senior career in team sport is affected by relative age effect. Frontiers in Psychology. 2019;10(1465):1–6. 31293489 10.3389/fpsyg.2019.01465 PMC6606777

[15] Votteler A, Höner O. The relative age effect in the German Football TID Programme: Biases in motor performance diagnostics and effects on single motor abilities and skills in groups of selected players. European Journal of Sport Science. 2014;14(5):433–42. 10.1080/17461391.2013.837510 24047192

[16] Till K, Cobley S, Wattie N, O’Hara J, Cooke C, Chapman C. The prevalence, influential factors and mechanisms of relative age effects in UK Rugby League. Scandinavian Journal of Medicine and Science in Sports. 2010;20(2):320–9. 10.1111/j.1600-0838.2009.00884.x 19486487

[17] Tribolet R, Watsford ML, Coutts AJ, Smith C, Fransen J. From entry to elite: The relative age effect in the Australian football talent pathway. Journal of Science and Medicine in Sport [Internet]. 2019;22(6):741–5. Available from: 10.1016/j.jsams.2018.12.014 30598253

[18] Nakata H, Sakamoto K. Relative Age Effect in Japanese Male Athletes. Perceptual and Motor Skills. 2011;113(2):570–4. 10.2466/05.10.11.PMS.113.5.570-574 22185072

[19] Lidor R, Arnon M, Maayan Z, Gershon T, Côté J. Relative age effect and birthplace effect in Division 1 female ballgame players-the relevance of sport-specific factors. International Journal of Sport and Exercise Psychology. 2014;12(1):19–33.

[20] Smith KL, Weir PL, Till K, Romann M, Cobley SP. Relative age effects across and within female sport contexts: A systematic review and meta-analysis. Sports Medicine [Internet]. 2018;48(6):1451–78. Available from: 10.1007/s40279-018-0890-8 29536262

[21] Sedano S, Vaeyens R, Redondo JC. The Relative Age Effect in Spanish Female Soccer Players. Influence of the Competitive Level and a Playing Position. Journal of Human Kinetics. 2015;46(1):129–37. 26240656 10.1515/hukin-2015-0041 PMC4519203

[22] Schorer J, Baker J, Lotz S, Büsch D. Influence of early environmental constraints on achievement motivation in telented young handball players. International Journal of Sport Psychology. 2010;41:42–58.

[23] Schorer J, Wattie N, Baker J. A new dimension to relative age effects: constant year effects in German youth handball. PLoS ONE. 2013;8(4):1–7. 10.1371/journal.pone.0060336 PMC3637174 23637745

[24] Sánchez-Rodríguez C, Grande I, Sampedro J, Rivilla J. Is the date of birth an advantage / ally to excel in handball? Journal of Human Sport and Exercise. 2013;8:2–5.

[25] Gómez-López M, Granero-gallegos A, Feu-Molina S, Luis J. Relative age effect during the selection of young handball player. Journal of Physical Education and Sport. 2017;17(1):418–23.

[26] Wrang CM, Rossing NN, Diernæs RM, Hansen CG, Dalgaard-Hansen C, Karbing DS. Relative Age Effect and the Re-Selection of Danish Male Handball Players for National Teams. Journal of Human Kinetics. 2018;63(1):33–41. 30279939 10.2478/hukin-2018-0004 PMC6162975

[27] Bjørndal CT, Luteberget LS, Till K, Holm S. The relative age effect in selection to international team matches in Norwegian handball. PLoS ONE [Internet]. 2018;13(12):1–12. Available from: https://www.scopus.com/inward/record.uri?eid=2-s2.0-85058848172&doi=10.1371%2Fjournal.pone.0209288&partnerID=40&md5=edd4c7706c59bc88a1d197c618853a96 10.1371/journal.pone.0209288 PMC6300244 30566450

[28] Fonseca FS, Figueiredo LS, Gantois P, de Lima-Junior D, Fortes LS. Relative age effect is modulated by playing position but is not related to competitive success in elite under-19 handball athletes. Sports [Internet]. 2019 4 19;7(91):1–10. Available from: https://www.mdpi.com/2075-4663/7/4/91 10.3390/sports7040091 PMC6524358 31010139

[29] Collins D, Bailey R. ‘Scienciness’ and the allure of second- hand strategy in talent identification and development. International Journal of Sport Policy and Politics. 2013;5(2):183–91.

[30] Cobley SP, Schorer J, Baker J. Relative age effects in professional German soccer: A historical analysis. Journal of Sports Sciences. 2008;26(14):1531–8. 10.1080/02640410802298250 19040189

[31] Rea LM, Parker RA. Design and conducting survey research. A comprehensive guide Jossey-Bass Publishers, editor. San Francisco; 1992.

[32] Delorme N, Boiché J, Raspaud M. The relative age effect in elite sport: The French case. Research Quarterly for Exercise and Sport. 2009;80(2):336–44. 10.1080/02701367.2009.10599568 19650399

[33] Brazo-Sayavera J, Martínez-Valencia MA, Müller L, Andronikos G, Martindale RJJ. Relative age effects in international age group championships: A study of Spanish track and field athletes. PLoS ONE. 2018;13(4):1–11. 10.1371/journal.pone.0196386 PMC5916855 29689117

[34] Romann M, Cobley S. Relative age effects in athletic sprinting and corrective adjustments as a solution for their removal. PLoS ONE. 2015;10(4):1–12. 10.1371/journal.pone.0122988 PMC4386815 25844642

[35] Brustio PR, Lupo C, Ungureanu AN, Frati R, Rainoldi A, Boccia G. The relative age effect is larger in Italian soccer top-level youth categories and smaller in Serie A. PLoS ONE. 2018;13(4):1–12. 10.1371/journal.pone.0196253 PMC5909613 29672644

[36] Massuça LM, Fragoso I, Teles J. Attributes of Top Elite Team-Handball Players. Journal of Strength and Conditioning Research [Internet]. 2014 1;28(1):178–86. Available from: https://insights.ovid.com/crossref?an=00124278-201401000-00024 10.1519/JSC.0b013e318295d50e 23591948

[37] Maroto-Izquierdo S, García-López D, de Paz JA. Functional and Muscle-Size Effects of Flywheel Resistance Training with Eccentric-Overload in Professional Handball Players. Journal of Human Kinetics. 2017;60(1):133–43. 29339993 10.1515/hukin-2017-0096 PMC5765793

[38] Mohamed H, Vaeyens R, Matthys S, Multael M, Lefevre J, Lenoir M, et al Anthropometric and performance measures for the development of a talent detection and identification model in youth handball. Journal of Sports Sciences. 2009;27(3):257–66. 10.1080/02640410802482417 19153859

[39] Schorer J, Cobley S, Büsch D, Bräutigam H, Baker J. Influences of competition level, gender, player nationality, career stage and playing position on relative age effects. Scandinavian Journal of Medicine and Science in Sports. 2009;19(5):720–30. 10.1111/j.1600-0838.2008.00838.x 18627551

[40] Goldschmied N, Cobley SP, Wattie N, Baker J, McKenna J. No Evidence for the Relative Age Effect in Professional Women ‘ s Sports Authors ‘ Reply Relative Age Effects in Female. Sports Medicine. 2011;41(1):87–90. 10.2165/11586780-000000000-00000 21142286

[41] Helsen WF, Hodges NJ, Van Winckel J, Starkes JL. The roles of talent, physical precocity and practice in the development of soccer expertise. Journal of Sports Sciences. 2000;18(9):727–36. 10.1080/02640410050120104 11043898

[42] Leite N, Borges J, Santos S, Sampaio J. The relative age effect in school and federative sport in basketball. Revista de Psicología del Deporte. 2013;22(1):219–23.

[43] Gómez-López M, Sánchez SA, Granero-Gallegos A, Chirosa Ríos LJ. Relative age effect in handball players of Murcia: Influence of sex and category of game. Journal of Human Sport and Exercise. 2017;12(3):565–73.

[44] Aguilar OG, García MS, Marín JC, Romero JJF. Influence of a player’s year of birth on the chances of being talent-spotted in international women’s handball. Apunts Educacion Fisica y Deportes. 2012;108(2):54–60.

[45] Baker J, Cobley SP, Winckel V. Gender, depth of competition and relative age effects in team sports. Asian Journal of Exercise & Sports Science. 2009;6(1):1–8.

[46] Delorme N, Boiché J, Raspaud M. Relative age effect in elite sports: Methodological bias or real discrimination? European Journal of Sport Science. 2010;10(2):91–6.

[47] Fumarco L, Gibbs BG, Jarvis JA, Rossi G. The relative age effect reversal among the National Hockey League elite. PLoS ONE. 2017;12(8):1–16. 10.1371/journal.pone.0182827 PMC5555707 28806751

[48] McCarthy N, Collins D. Initial identification & selection bias versus the eventual confirmation of talent: evidence for the benefits of a rocky road? Journal of Sports Sciences [Internet]. 2014;32(17):1604–10. Available from: 10.1080/02640414.2014.908322 24857164

[49] Till K, Cobley SP, Morley D, O’Hara J, Chapman C, Cooke C. The influence of age, playing position, anthropometry and fitness on career attainment outcomes in rugby league. Journal of Sports Sciences [Internet]. 2016;34(13):1240–5. Available from: 10.1080/02640414.2015.1105380 26512761

[50] McCarthy N, Collins D, Court D. Start hard, finish better: further evidence for the reversal of the RAE advantage. Journal of Sports Sciences [Internet]. 2016;34(15):1461–5. Available from: 10.1080/02640414.2015.1119297 26651240

[51] Schorer J, Baker J, Büsch D, Wilhelm A, Pabst J. Relative age, talent identification and youth skill development: Do relatively younger athletes have superior technical skills? Talent Development & Excellence. 2009;1(1):45–56.

[52] Baker J, Horton S. A review of primary and secondary influences on sport expertise. High Ability Studies. 2004;15(2):211–28.

[53] Wattie N, Cobley S, Macpherson A, Howard A, Montelpare WJ, Baker J. Injuries in Canadian youth ice hockey: The influence of relative age. Pediatrics. 2007;120(1):1–7. 10.1542/peds.2006-1465 17606571

[54] Ashworth J, Heyndels B. Selection bias and peer effects in team sports. The effect of age grouping on earnings of German soccer players. Journal of Sports Economics. 2007;8(4):355–77.

[55] Yagüe JM, de la Rubia A, Sánchez-Molina J, Maroto-Izquierdo S, Molinero O. The Relative Age Effect in the 10 Best Leagues of Male Professional Football of the Union of European Football Associations (UEFA). Journal of sports science & medicine. 2018;17(3):409–16. 30116114 PMC6090398

[56] Kruger K, Pilat C, Uckert K, Frech T, Mooren FC. Physical performance profile of handball players is related to playing position and playing class. Journal of Strength and Conditioning Research. 2014;28(1):117–25. 10.1519/JSC.0b013e318291b713 23539084

[57] Massuca L, Branco B, Miarka B, Fragoso I. Physical Fitness Attributes of Team-Handball Players are Related to Playing Position and Performance Level. Asian Journal of Sports Medicine [Internet]. 2015 3 1;6(1):2–6. Available from: http://asjsm.com/en/articles/21620.html 10.5812/asjsm.24712 PMC4393545 25883775

[58] Nikolaidis PT, Ingebrigtsen J, Povoas SC, Moss S, Torres-Luque G. Physical and physiological characteristics in male team handball players by playing position—Does age matter? The Journal of sports medicine and physical fitness. 2015;55(4):297–304. 25303066

[59] Matthys S, Fransen J, Vaeyens R, Philippaerts R. Differences in biological maturation, anthropometry and physical performance between playing positions in youth team handball. Journal of Sports Sciences. 2013;31(12):1344–52. 10.1080/02640414.2013.781663 23656188

[60] Manchado C, Tortosa-Martinez J, Vila H, Ferragut C, Platen P. Performance factors in women’s team handball: Physical and Physiological aspect—A review. Journal of Strength and Conditioning Research. 2013;27(6):1708–19. 10.1519/JSC.0b013e3182891535 23439330

[61] Wattie N, Schorer J, Baker J. The relative age effect in sport: A developmental systems model. Sports Medicine. 2015;45(1):83–94. 10.1007/s40279-014-0248-9 25169442

[62] Barnsley R, Thompson A, Legault P. Family planning: football style. The relative age effect in football. International Review for the Sociology of Sport. 1992;27(1):77–87.

[63] Arrieta H, Torres-Unda J, Gil SM, Irazusta J. Relative age effect and performance in the U16, U18 and U20 european basketball championships. Journal of Sports Sciences. 2016;34(16):1530–4. 10.1080/02640414.2015.1122204 26666180

[64] Sarvestan J, Riedel V, Gonosová Z, Linduška P, Přidalová M. Relationship between anthropometric and strength variables and maximal throwing velocity in female junior handball players—a pilot study Relationship between anthropometric and strength variables and maximal throwing velocity in female junior handball p. Acta Gymnica. 2019;49(3):132–7.

[65] Zapartidis I, Skoufas D, Vareltzis I, Christodoulidis T, Toganidis T, Kororos P. Factors Influencing Ball Throwing Velocity in Young Female Handball Players. The Open Sports Medicine Journal. 2009;3(1):39–43.

[66] Tenenbaum G. Expert athletes: An integrated approach to decision making. Expert performance in sports. 2003;191–218.

[67] Werneck FZ, Coelho E., de Oliveira H.Z., Ribeiro-Júnior DB, Almas SP, de Lima JRP, et al Relative age effect in Olympic basketball athletes. Science & Sports [Internet]. 2016;31(3):158–61. Available from: 10.1016/j.scispo.2015.08.004

[68] Kirkendall DT. The relative age effect has no influence on match outcome in youth soccer. Journal of Sport and Health Science [Internet]. 2014;3(4):273–8. Available from: 10.1016/j.jshs.2014.07.001

[69] Hancock DJ, Adler AL, Côté J. A proposed theoretical model to explain relative age effects in sport. European Journal of Sport Science. 2013;13(6):630–7. 10.1080/17461391.2013.775352 24251740

[70] Cobley SP, Till K. Participation trends according to relative age across youth UK Rugby League. International Journal of Sports Science & Coaching. 2017;12(3):339–43.

[71] Abbott A, Button C, Zealand N, Collins D. Unnatural Selection: Talent Identification and Development in Sport. Nonlinear Dynamics, Psychology, and Life Sciences. 2005;9(1):61–88. 15629068

[72] Bjørndal CT, Ronglan LT. Engaging with uncertainty in athlete development-orchestrating talent development through incremental leadership. Sport, Education and Society [Internet]. 2019;1–13. Available from: 10.1080/13573322.2019.1695198

[73] Pearson DT, Naughton GA, Torode M. Predictability of physiological testing and the role of maturation in talent identification for adolescent team sports. Journal of Science and Medicine in Sport. 2006;9(4):277–87. 10.1016/j.jsams.2006.05.020 16844415

[74] Burgess DJ, Naughton GA. Talent development in adolescent team sports: A review. International Journal of Sports Physiology and Performance. 2010;5(1):103–16. 10.1123/ijspp.5.1.103 20308701

[75] Lidor R, Côté J, Hackfort D. ISSP position stand: To test or not to test? The use of physical skill tests in talent detection and in early phases of sport development. International Journal of Sport and Exercise Psychology. 2009;7(2):131–46.

[76] Vaeyens R, Matthieu L, Williams MA, Philppaerts R. Talent Identification and Development Programmes in Sport. Sports Medicine. 2008;38(9):703–14. 10.2165/00007256-200838090-00001 18712939

[77] Andersen SS, Bjørndal CT, Ronglan LT. The ecology of talent development in Nordic elite sport model. In: Routledge, editor. Managing Elite Sport Systems Research and Practice. Routledge; 2015 p. 61–78.

